# Misinformation on social networks during the novel coronavirus pandemic: a quali-quantitative case study of Brazil

**DOI:** 10.1186/s12889-021-11165-1

**Published:** 2021-06-23

**Authors:** Priscila Biancovilli, Lilla Makszin, Claudia Jurberg

**Affiliations:** 1 Doctoral School of Health Sciences, University of Pécs, Pécs, Hungary; 2 Institute of Bioanalysis, Medical School, University of Pécs, Pécs, Hungary; 3 Szentágothai Research Center, University of Pécs, Pécs, Hungary; 4 Federal University of Rio de Janeiro, Oncobiology Program, Institute of Medical Biochemistry Leopoldo de Meis, Rio de Janeiro, Brazil; 5 Oswaldo Cruz Foundation, Oswaldo Cruz Institute, Rio de Janeiro, Brazil

**Keywords:** COVID-19, Coronavirus, Misinformation, Social media, Politics, Brazil, Pandemic, Fact check

## Abstract

**Background:**

One of the challenges posed by the novel coronavirus pandemic is the *infodemic risk*, that is, a huge amount of information being published on the topic, along with misinformation and rumours; with social media, this phenomenon is amplified, and it goes faster and further. Around 100 million people in Brazil (50% of the inhabitants) are users of social media networks – almost half of the country’s population. Most of the information on the Internet is unregulated, and its quality remains questionable.

**Methods:**

In this study, we examine the main characteristics of misinformation published on the topic. We analysed 232 pieces of misinformation published by the Brazilian fact-checking service “Agência Lupa”. The following aspects of each news item were analysed: a) In what social media has it circulated?; b) What is the content classification, sentiment and type of misinformation?; d) Are there recurrent themes in the sample studied?

**Results:**

Most were published on Facebook (76%), followed by WhatsApp, with 10% of total cases. Half of the stories (47%) are classified as “real-life”, that is, the focus is on everyday situations, or circumstances involving people. Regarding the type of misinformation, there is a preponderance of fabricated content, with 53% of total, followed by false context (34%) and misleading content (13%). Wrong information was mostly published in text format (47%). We found that 92.9% of misinformation classified as “fabricated content” are “health tips”, and 88.9% of “virtual scams” are also fabricated.

**Conclusion:**

Brazilian media and science communicators must understand the main characteristics of misinformation in social media about COVID-19, so that they can develop attractive, up-to-date and evidence-based content that helps to increase health literacy and counteract the spread of false information.

## Background

The novel coronavirus (SARS-CoV-2) pandemic generated an avalanche of information that circulates daily around the world. Throughout 2020, millions of people were quarantined due to the pandemic and suffered negative psychological effects, including post-traumatic stress symptoms, confusion, and anger [1]. The Internet is the easiest and fastest source to obtain health information in this context [2]. A public health crisis that involves numerous uncertainties requires precise information and answers for the adoption of appropriate behaviours and intelligent decision making [3].

One of the challenges posed by this new coronavirus is the *infodemic risk*, that is, a tsunami of information about the topic that can also bring rumours and misinformation; with social media, this phenomenon is amplified, and it goes faster and further [4]. A study that investigated true and false news stories on the Twitter network concluded that falsehood was spread significantly faster and more broadly than the truth in all categories of information [5]. For this reason, it is important that the population is not only informed in real time but that information needs to be correct and updated, so as many people as possible can act properly to avoid spreading the disease.

### The context of the study: the novel coronavirus pandemic in Brazil

The novel coronavirus in Brazil arrived in a scenario of a conservative, far-right government led by President Jair Bolsonaro, which systematically denies the severity of the SARS-CoV-2 pandemic [6]. The attitude of the president and his ministers drew severe criticism from the international press [7–9]. Moreover, the World Health Organization (WHO) [10] also criticised Brazil’s stance in controlling the pandemic, and for that reason the president threatened to pull Brazil out of the institution [11].

The protective guidelines announced by the World Health Organization and endorsed by the Ministry of Health were routinely questioned by President Bolsonaro. In addition to it, Bolsonaro has encouraged people to go out and even make appearances in stores, markets and public demonstrations on the streets [6]. He also supported the use of anti-malarial drugs as a ‘preventive kit’ to avoid COVID-19 infection [12]. In addition to the president’s negationist attitudes towards the virus, WHO guidelines have not always been consistent. For example, in January 2020, WHO guidance stated that only people with flu symptoms should use medical masks [13]; over the months, they began to recommend the use of masks by the entire population, as many asymptomatic or pre-symptomatic patients can still infect other people [14]. This caused misunderstandings about the seriousness of the pandemic, not only in Brazil but also in other countries [15]. As of May 12th 2021, Brazil had registered more than 15.1 million cases of the novel coronavirus, and the number of fatal cases had surpassed 422.3 thousand [16].

In Brazil, about 100 million people are users of social networks [17]. This number corresponds to almost half of the country’s total population [18]. Online access to health information has been growing exponentially in the last few years. However, most of the information on the Internet is unregulated [19]. New media platforms are less formally governed in many instances than broadcast mass media content, and online content creators can spread some types of misinformation that would be protected as free speech [20].

### Definition and types of misinformation

Misinformation on the Internet started to attract the attention of the media and academics during the U.S. elections in 2016; at that time, the expression “fake news” became increasingly popularised [21]. Despite the extensive use of this term in the media and in scientific articles, it is considered inadequate to capture the complexity of the information disorder phenomenon [22–24]. Fake news coincides with other information disorders, such as misinformation (false, mistaken or misleading information) and disinformation (false information that is purposely spread to deceive or confuse people) [24]. Moreover, some authors suggest that the fake news label is used as a political instrument to discredit legacy news media, which can decrease citizens’ levels of media trust [25–27].

Even considering these differences, it is not always easy to fit a news item into misinformation/disinformation categories, because we do not always know if the author of the news had the deliberate intention to deceive, or if he/she really believes in what is being written. For this reason, we follow the same classification as Wang et al. [23], which uses misinformation as an umbrella term that encompasses all types of false health information, unless the intention to deceive is evident.

The combination of *infodemic* brought about by the novel coronavirus disease situation, with the considerable presence of Brazilians on social networks, many of them without the full capacity to discern the quality of what is published, brings up a potential risk to public health in the country [28]. The flood of misinformation and manipulated information on social media should be recognised as a global public health threat [28, 29]. Misinformation is concerning because of its potential to influence attitudes and behaviour, which can have negative consequences for social harmony and health, among other aspects [30]. Given this context, the aim of this study is to understand the format and content characteristics of Brazilian misinformation that circulates on social media. We try to answer the following research questions: **RQ1:** What kind of content, type of rumour, sentiments, and multimedia types are more frequent? **RQ2:** In which social networks is misinformation about COVID-19 in Brazil propagated the most? **RQ3:** Is the misinformation curve on social networks about the novel coronavirus in Brazil directly proportional to the increase in the number of cases in the country, reflecting the growing interest of the population on the topic as the virus spreads?

## Methods

This is a quali-quantitative exploratory case study. We analysed all pieces of misinformation published by the Brazilian fact-checking service Agência Lupa (Lupa agency, in English) in the first 27 weeks (six months) of 2020 (from January 1, 2020 to July 4, 2020). Lupa agency was created in 2015 and is the first company specialised in fact-checking in Brazil; the checking is carried out by specialised journalists, based on successful processes implemented by fact-checking platforms such as the Argentine Chequeado and the North American Politifact [31]. This agency is part of the International Fact-Checking Network (IFCN), a worldwide network dedicated to bringing together fact-checkers worldwide; as a “verified member” of IFCN, the agency undergoes independent audits every year [31].

As described on the website of Lupa agency [32], the production process begins with the selection of content that can be fact-checked. For this, Lupa journalists investigate daily what is said by politicians, social leaders and celebrities, in newspapers, magazines, radio, TV shows and on the internet. When selecting the content they want to work on, the team adopts three relevance criteria. They give preference to statements made by prominent national figures, to matters of public interest and / or that have recently gained prominence in the press or on the internet. In addition, they are also dedicated to debunking, which is the verification of content published by unofficial sources. The content is checked if it has historical data, or statistical data, or comparisons and statements about the legality of a fact [33]. After deciding the content to be fact-checked, the agency’s journalists collect newspapers, magazines and websites that address the topic, interview specialists or even investigate the news in person, if possible [32].

All news related to the novel coronavirus in the period was organised in an Excel table. For a news item to be considered as related to the topic, the fact-checking text should have at least once the terms “coronavirus” or “COVID-19”. The term “pandemic” is never mentioned alone in the news stories of Lupa Agency, therefore we decided to not use it in our search. The following aspects of each news item were analysed: a) In what social media has it circulated?; b) What is the content classification, sentiment and type of misinformation?; d) Are there recurrent themes in the sample studied?

### Misinformation, content and sentiment analysis of news stories

The nomenclature developed by Wardle [24] on the different types of misinformation inspired this data analysis. We used the following categories: a) **Misleading content** describes stories which are not entirely false but lead the reader to misinterpret the data; b) **Fabricated content** refers to 100% false pieces of information, with nothing that can be assessed as true; c) **False context** encompasses Wardle’s categories of false context, false connection and manipulated content. The news was classified like this when headlines, visuals or captions do not support the content, or when genuine information (texts, photos or videos) are manipulated; d) **Satire or parody** refers to news that are not meant to be taken seriously, as the main motivation is comical. For this analysis, as in Sommariva et al. [34] we decided not to include the category **imposter content,** when genuine sources are impersonated. This is because the analysis of misinformation producers is not within the scope of this study.

The content analysis of the texts was based on the methodology proposed by Laurence Bardin [35], which is an inductive process. Firstly, two researchers read in depth (and independently) a sample of 20 news stories. Then, based on the readings, each researcher created a list of categories to describe them. Categorisation followed a semantic criterion: the news was separated according to the theme, and they could not be classified in more than one category. At the same time, the sentiment analysis of each text was also made. Sentiment analysis is the task of identifying positive and negative opinions, emotions, and evaluations [36]. Two researchers read all texts and identified the predominant sentiment (positive, negative or neutral) through document-level analysis [37]. The task at this level is to determine the overall opinion of the document. Sentiment analysis at document level assumes that each document expresses opinions on a single entity [38].

Percent agreement was used to calculate intercoder reliability, and the result was 81%. Before classifying the full sample, the authors discussed their experiences and achieved consensus regarding inconsistencies. Then, one author coded the remaining messages.

### Statistical analyses

All statistical analyses were performed using SPSS Statistics Version 26.0 (IBM, 2020) and Microsoft Office Excel Version 16 (USA, 2018). Fisher’s Exact Test is used to determine the relationship between two categorical variables; this test is the only good option to check if there are significant relationships between two categorical values on a small sample. *P*-values less than 0.05 are considered statistically significant.

## Results

### Content and media where misinformation was found

**Fig. 1:**
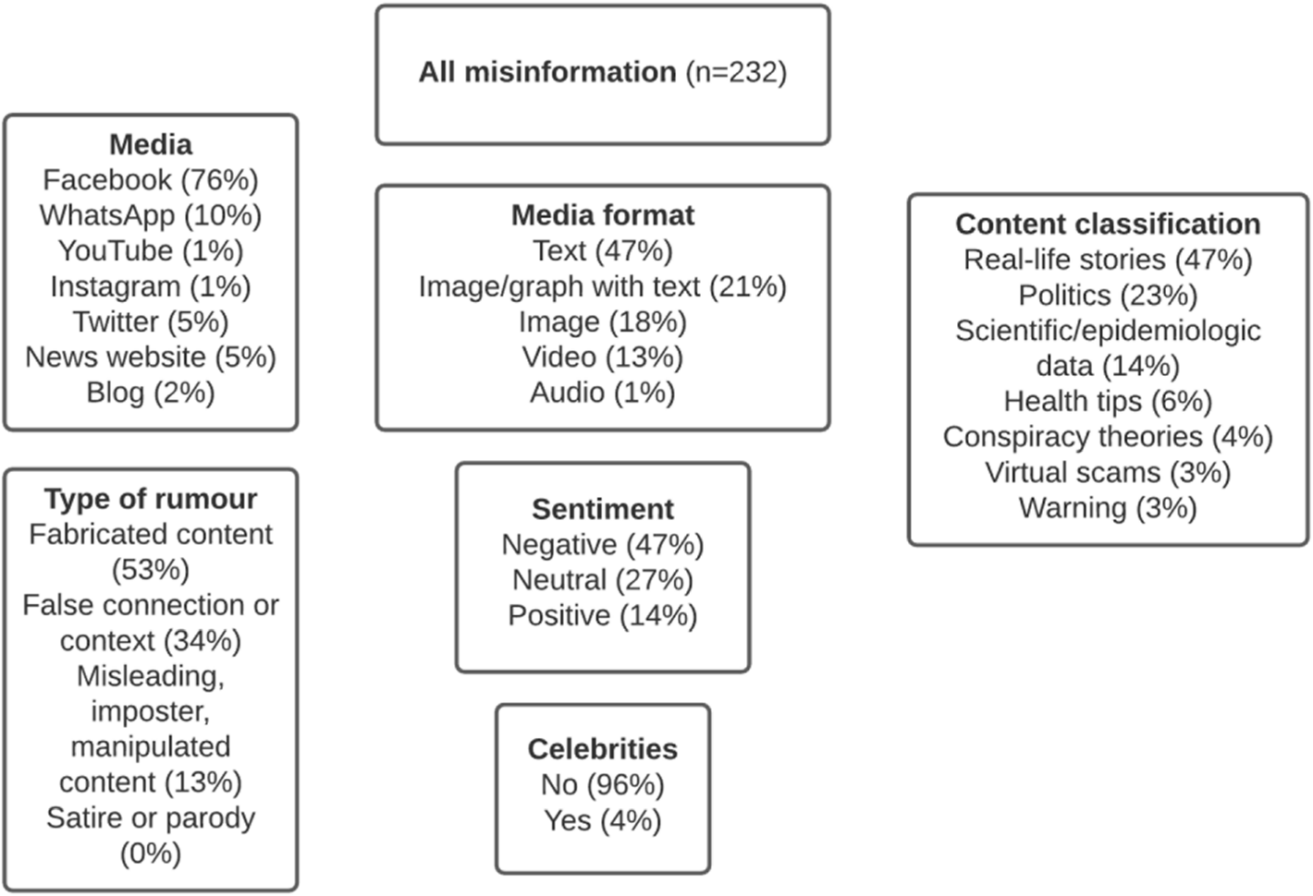
Characteristics of misinformation about COVID-19 in Brazil, between January 1, 2020 and July 4, 2020

A total of 232 pieces of misinformation were analysed, starting from **week 4** of 2020, when the first fact-checked story was published and finishing at week 27 of 2020. Regarding **RQ1**, we can see a prevalence of certain misinformation contents in Brazil, as shown in Fig. 1. The stories were classified into seven content categories. Some categories are similar to other studies that analysed discourses in social networks [39, 40]. We can observe that almost half of the stories (47%) are classified as “real-life”, that is, the focus is on everyday situations, or situations involving people.

The second most frequent category is “politics”. Here are the stories that focus on politicians, governments, parties, political decisions, aid from governments, laws, decrees, or messages of adulation/adoration aimed at a politician, and they represent 23% of the total amount of registered news. In third place is information on advances in “science and epidemiological data” (14%). Pieces of misinformation related to virtual scams, conspiracy theories and warnings (any kind of warning about what to do or not to do during the pandemic) account for 10% of the total, followed by “health tips” (6%).

There was a very strong connection between media format and type of rumour (*p* < 0.001; Cramer’s V value = 0.314). Regarding “fabricated content”, it is important to note that 89.7% of misinformation has text as media format, 68.3% has image/graph with text, and 45.3% has image only, whereas “false content” shows 74.1% video, 40% image, and 19.5% image/graph with text.

We also identified a strong connection between media and type of rumour, with “fabricated content” being more frequent on WhatsApp, while “false connection” is more frequent on YouTube (*p* = 0.002; Cramer’s value = 0.215). There is a very strong connection between content classification and sentiment, (*p* < 0.001; Cramer’s value = 0.319), as well as between content classification and type of rumour (*p* < 0.001; Cramer’s value = 0.402).

Regarding the relationship between content classification and type of rumour (see Table 1), we found that 92.9% of misinformation classified as “fabricated content” are “health tips”, and 88.9% of “virtual scams” are also fabricated. “Fabricated content” is the most frequent type of rumour across almost all content types, except “real-life stories”. In this case, “false connection or false context” is the type of rumour with most cases - 57.4% of the total. Concerning “scientific/epidemiologic data”, 43.8% of the stories fall into “misleading, imposter, manipulated content” type of rumour.

**Table 1:** Relationship between content classification and type of rumour among all 232 pieces of misinformation analysed

			Real life stories	Conspiracy theories	Health Tips	Scientific/epidemiologic data	Virtual scams	Warnings	Politics	Total
Type of rumour	Satire or Parody	Frequency	0	0	0	0	1	0	0	1
		%	0,0%	0,0%	0,0%	0,0%	11,1%	0,0%	0	0,4%
		Adjusted Residual	-,9	-,2	-,2	-,4	5,1	-,2	-,5	
	Misleading, imposter, manipulated content	Frequency	5	1	1	14	0	2	3	26
		%	4,3%	10,0%	7,1%	43,8%	0,0%	28,6%	5,4%	10,7%
		Adjusted Residual	−3,0	-,1	-,4	6,5	−1,1	1,6	−1,5	
	Fabricated content	Frequency	44	7	13	17	8	5	36	130
		%	38,3%	70,0%	92,9%	53,1%	88,9%	71,4%	64,3%	53,5%
		Adjusted Residual	−4,5	1,1	3,0	,0	2,2	1,0	1,8	
	False connection or false context	Frequency	66	2	0	1	0	0	17	86
		%	57,4%	20,0%	0,0%	3,1%	0,0%	0,0%	30,4%	35,4%
		Adjusted Residual	6,8	−1,0	−2,9	−4,1	−2,3	−2,0	-,9	
Total		Frequency	115	10	14	32	9	7	56	243
		%	100,0%	100,0%	100,0%	100,0%	100,0%	100,0%	100,0%	100,0%

When we observe the relationship between content classification and sentiment (Table 2), we can see that 90% of the pieces of misinformation classified as “conspiracy theories” are considered to be negative, as well as 85.7% of “warnings” and 76.8% of stories classified as “politics”. We can also note that most (64.3%) of “health tips” have a neutral tone, as well as “virtual scams” (55.6%). None of the misinformation categories had a preponderance of positive sentiment; but 35.7% of “health tips” were positive, 33.3% of “virtual scams” and 25% of “scientific/epidemiologic data”.

**Table 2:** Relationship between sentiment and content classification among all 232 pieces of misinformation analysed

			Real life stories	Conspiracy theories	Health Tips	Scientific/epidemiologic data	Virtual scams	Warnings	Politics	Total
Sentiment	Negative	Frequency	75	9	0	13	1	6	43	147
		%	65,2%	90,0%	0,0%	40,6%	11,1%	85,7%	76,8%	60,5%
		Adjusted Residual	1,4	−1,9	−4,8	−2,5	−3,1	1,4	2,8	
	Neutral	Frequency	25	1	9	11	5	1	9	61
		%	21,7%	10,0%	64,3%	34,4%	55,6%	14,3%	16,1%	25,1%
		Adjusted Residual	−1,1	− 1,1	3,5	1,3	2,1	-,7	−1,8	
	Positive	Frequency	15	0	5	8	3	0	4	35
		%	13,0%	0,0%	35,7%	25,0%	33,3%	0,0%	7,1%	14,4%
		Adjusted Residual	-,6	−1,3	2,3	1,8	1,6	−1,1	− 1,8	
Total		Frequency	115		14	32	9	7	56	243
		%	100,0%	10	100,0%	100,0%	100,0%	100,0%	100,0%	100,0%

It is important to note that all misinformation published on news websites and blogs was also posted on Facebook. When the news appears simultaneously on Facebook and a blog or on Facebook and a news website, it means that there is a link in the Facebook post leading to an external page. In such cases, Facebook serves as a call for the reader to see the full story at the indicated link. In this way, we conclude that 100% of the fact-checked content was published on at least one social network.

It is interesting to note that the negative sentiment became predominant throughout the course of the epidemic in Brazil and was mainly associated with “real-life stories” and “politics”. Almost 50% of the information with a negative frame was concentrated on these two themes. Another curious fact was the low use (10/232 or 4%) of celebrities to disseminate false information.

Concerning **RQ2**, most pieces of misinformation were published on Facebook (76%), followed by WhatsApp, with 10% of total cases (see Fig. 1). This data sample does not correspond to the ranking of the most used social media platforms in Brazil. According to a recent survey conducted on the habits of Brazilians on social networks (We are Social, 2020), YouTube is the most accessed social media page (96% of Internet users aged 16 to 64 reported using this platform in December 2019); in second place is Facebook (90%), and in third place is WhatsApp (88%), followed by Instagram (79%), Facebook Messenger (66%) and Twitter (48%).

Although YouTube is the most popular social media by Internet users in Brazil, only three pieces of misinformation about COVID-19 were found there during this period. Moreover, Instagram do not seem to be popular social network in Brazil for the spread of misinformation, as only 1% of the total sample was found there.

### Misinformation about COVID-19 over time and compared to other topics

**Fig. 2:**
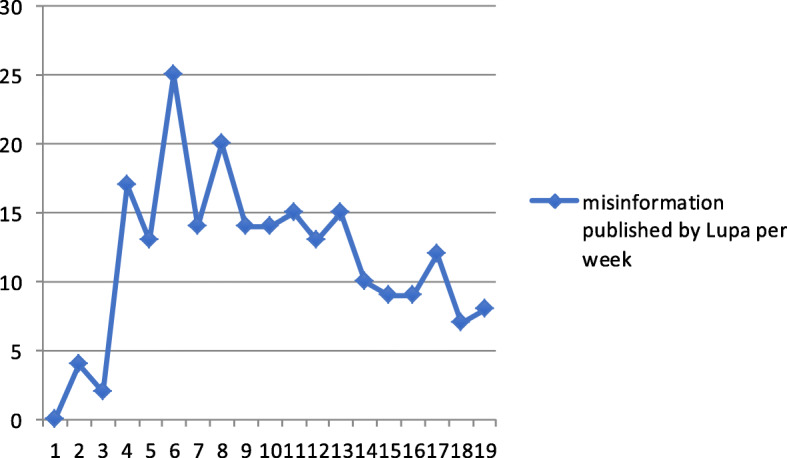
Number of fact-checked misinformation items detected by Lupa agency, since the first case recognised on February 26, 2020 (week 9) until the week 27, in July 4, 2020

**Fig. 3:**
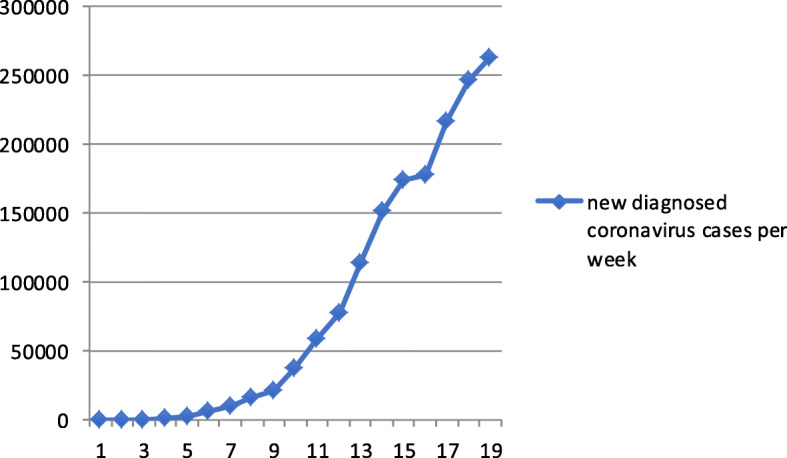
Number of new diagnosed coronavirus cases per week in the same period

Concerning **RQ3**, the first news item recognised by Lupa agency as false in Brazil dates to January 24, 2020 (week 4 of the year). In this week, only one piece of misinformation went through the fact-check process. The peak of fact-checked news occurred in week 14, when 24 pieces of misinformation were published. Figures 2 and 3 compare the number of diagnosed coronavirus cases per week with the number of pieces of misinformation detected by Lupa agency in the same period. We can see that there is no parallelism between the two phenomena.

## Discussion

This study explored the spread of misinformation on COVID-19 in Brazil through social media, analysing the stories published by the fact-checking service Lupa agency from January 1, 2020 to July 4, 2020. This is the first analysis of multiple characteristics of misinformation that circulate on Brazilian social media about the novel coronavirus. Although at the end of our sample the pandemic has not yet ended, this study aims to understand the flow of misinformation produced in the first half of 2020, the topics addressed in the country and how the media may reflect the positions of social actors and politicians that are relevant in Brazilian society at this time. Brazil is one of the countries that had an exponential increase in the disease case curve during the first half of 2020, and the situation became even more critical in the first four months of 2021, with a resurgence of COVID-19 cases in several Brazilian urban centres, especially Manaus, the capital of the state of Amazonas [41]. This could be due to the emergence of a new virus variant, named P.1, which is around 2.5 times more transmissible than that of the first wave [42]. The emergence of new, more transmissible variants [43] coupled with the slow vaccine rollout caused a brutal surge in deaths in the first months of 2021, reaching over 4000 fatalities/day in early April [12].

We can see that 100% of the pieces of misinformation in our sample were published on social networks. Even those on blogs and news websites were also posted (via hyperlink) on Facebook, which demonstrates the strength of these new media in spreading information. Social media play a vital role during crises, serving as both a first-hand information channel on the scene as well as a supplementary channel offering specific information demanded by people directly involved in the crisis [3]. However, during the pandemic, the frequent use of social networks is associated with a greater belief in false information [44].

Misinformation can have serious consequences for the population, affecting people’s knowledge, beliefs and memory [45]. Mistaken health tips or false scientific and epidemiological data can make people believe that the disease is not so serious, or that a few simple actions (such as drinking tea or gargling) are enough to prevent COVID-19. As this disease is extremely contagious [46], a less concerned behaviour of the population in relation to it can cause a loosening in measures of social distancing, which contributes to the faster spread of the virus [47] and the overwhelming of hospitals [48].

When we analyse the misinformation topics in our sample, we observe some specific themes. Fabricated news classified as “real-life stories” and “politics” were the most prevalent in the period. Within these topics, three subjects drew attention: a) Field hospitals supposedly being empty, which proves that the disease is not real (***n*** **= 14**); b) People cured by chloroquine and hydroxychloroquine (***n*** **= 13**); c) Burial of empty coffins as if they were patients killed by the virus (***n*** **= 7**).

The discussion about supposedly empty hospitals gained prominence when the curve of cases of the disease in Brazil began to accelerate, in April 2020 [49]. Some of the misinformation shows people filming or taking pictures of hospitals with empty receptions. What happens is that many of the hospitals receive only patients referred from other health units, which is why they do not perform emergency care. There are also videos that are contextually false, stating that there are many empty hospital beds, when in fact these videos are old or were filmed in other hospitals in smaller cities. In an official speech after the disclosure of these false news items, Bolsonaro asked supporters to enter hospitals to film whether beds are really occupied [50], which goes against the guidance of doctors due to the risk of contagion. In fact, some of his followers followed his request and invaded hospitals with patients hospitalised for the novel coronavirus, speaking loudly and disrespecting the medical team [51].

Regarding the news involving coffins, one of the pieces of misinformation that most caught the public’s attention was one that said that “coffins of victims of COVID-19 in Belo Horizonte were full of stones”. Another report states that “pits were opened to bury empty coffins in Marabá”. Such misinformation gained prominence on social networks and became a subject in society. For this reason, families began to gather to open sealed coffins with victims of COVID-19, to check if the body they were about to bury was really their family member [52]. In one case, five people were infected due to this action [53].

In our sample, 13 of the 232 pieces of misinformation address chloroquine or hydroxychloroquine. All stories treat these drugs as a cure for COVID-19, some with testimonies from celebrities (Tom Hanks’s wife) or anonymous people, and others condemning politicians for not believing in the power of those medicines. Mr. Bolsonaro defended the use of hydroxychloroquine against COVID-19 in a pronouncement broadcast on national television in April 2020 [54]. However, neither lab-based studies nor clinical trials have provided convincing evidence to support the value of hydroxychloroquine in the treatment of this disease [55, 56]. In other Latin American countries, such as Dominican Republic, clients without a prescription purchase these drugs, as there is a culture of self-medication and lack of governmental regulation on drug use [57]. This same culture exists in all regions of Brazil.

Most of the news classified as “politics” has a negative sentiment (76.8%). The same trend was observed in a study dedicated to capture the main themes under discussion in the Brazilian media during the pandemic [58]. They observed that, on Twitter, themes classified as political, confirmed cases, prevention and control, and economic influences are positioned lower on the sentiment scale.

We see in many cases the construction of a narrative of denial of reality based on unrealistic arguments - as in the examples mentioned above. For the same reason, misinformation classified as “conspiracy theories” also has a mostly negative sentiment (90.0%). In relation to this category, we can observe narratives involving the Chinese government or Chinese citizens. For example, one of the stories states that Chinese researcher Bing-Liu, who was murdered in May in the United States, was about to create a vaccine for COVID-19. In fact, this computer scientist studied the COVID-19, but there is no evidence that the crime was committed because of this. Another piece of misinformation says that “China bought multinationals during the COVID-19 pandemic”. In fact, part of the shares of the companies mentioned (Volvo, Pirelli, Thomas Cook and Mercedes Benz) were bought by Chinese companies, but that was before the pandemic. This type of false information fuels hate speech against the Chinese population, to the point that they experience reduced self-acceptance, lower autonomy, compromised relationships, and other psychological effects, some of which could persist over time [59].

The simple fact that journalists dedicate part of their time to address and correct false information, not only at Lupa agency but also in other media, already makes the subject more widespread and, therefore, more debated. A network analysis concluded that the websites targeted with the most hyperlinks from fake news networks were mainstream media, social networking sites, and Wikipedia; few of the targeted websites linked back to the fake news sites [60].

The predominant frame in misinformation is negationist and endorsed by President Bolsonaro’s speeches, encouraging disrespectful, dangerous and even bizarre behaviour by part of the population. The very fact that the president ignores health recommendations for the pandemic sets a dangerous precedent, causing part of the population to do the same and thus increasing the contagion curve and the number of preventable deaths [61].

The actions of a political leader are essential to coordinate, organise and ensure that the rules are obeyed by citizens. In a pandemic, the need for a leader who recognises the seriousness of the problem and takes quick action is even greater. A study conducted during the novel coronavirus pandemic [6] showed that the social distancing measures taken by citizens in pro-government localities weakened compared to places where political support of the president is less strong; they also found evidence that this is stronger in municipalities with a larger proportion of Evangelical parishioners, a key group in terms of support for the president.

Another study that analysed the information disseminated by public health officials during the MERS outbreak in South Korea found that less credible information from those professionals led to more frequent use of online news and social media for acquiring information related to the disease [3].

In our sample, 43.8% of the pieces of misinformation classified as “misleading/imposter/manipulated” have a scientific/epidemiological content. An example is an image stating that “doctors from 30 countries confirm the effectiveness of chloroquine”, which is false. The post was based on the COVID-19 Real-Time Barometer, a weekly survey conducted by health platform Sermo [62]. Chloroquine and hydroxychloroquine were not considered the best treatment in the two most recent stages of the survey - this occurred only in the first survey, in March 2020. Another story mentions that the World Bank considered Brazil as the best country in the fight against COVID-19. The institution has never released a ranking on this topic. They actually praised the fact that Brazil maintained trade flows, which lessens the negative impact of the pandemic for the most vulnerable [63].

The guidelines of WHO leaders emphasise the importance of a broad and coherent response from Brazil, especially from governments (at the federal, state or municipal level), to control the pandemic [64]. The lack of a unified response makes the population confused, facilitating denialist attitudes, and neglecting individual actions to protect against the disease.

As a limitation of our study, we were unable to measure the public engagement of fact-checked stories, to get a more accurate idea of how many people were directly affected by the misinformation. In addition, we have no way of controlling how many people had access to or shared the pieces of misinformation circulating on WhatsApp, due to the private nature of their groups and the lack of data on engagement in this social network. Another limitation is the fact that we used the news verified by Lupa agency only as the basis for this research. As the amount of news circulating about the pandemic is enormous, we have no way of knowing whether the news verified in this period represents the entire sample of misinformation shared in Brazil. Despite these limitations, we believe that this work can offer help so that scientists, journalists and health educators understand the characteristics of the misinformation health agenda during the pandemic and are better able to counter this problem.

## Conclusions

In face of all the challenges discussed in this article, the Brazilian media and science communicators must understand the main characteristics of misinformation in social media about COVID-19, so that they can develop evidence-based digital policy action plans that helps to increase health literacy and modulate the perception of risk. Misinformation found in our sample contradict the generally accepted consensus of the scientific community to combat the pandemic. Exposure to misinformation can affect people’s knowledge, beliefs and memory. Therefore, health educators must have a massive presence in the most popular social media sites with attractive, readable and up-to-date content, as an effort to counteract the spread of false information and the mistrust of public health institutions.

## Data Availability

The datasets used and/or analysed during the current study are available from the corresponding author on reasonable request.

## Abbreviations

COVID-19: 2019 novel coronavirus
SARS-CoV-2: Severe acute respiratory syndrome coronavirus 2
WHO: World Health Organization
U.S.: The United States of America
SPSS: Statistical Package for the Social Sciences
P: *p*-value
MERS: Middle East respiratory syndrome
